# Mathematical modeling in perspective of vector-borne viral infections: a review

**DOI:** 10.1186/s43088-022-00282-4

**Published:** 2022-08-19

**Authors:** Ramakant Prasad, Surendra Kumar Sagar, Shama Parveen, Ravins Dohare

**Affiliations:** 1 Department of Mathematics, Gargi College (University of Delhi), New Delhi, India; 2 Department of Zoology, Swami Sraddhanand College (University of Delhi), New Delhi, India; 3 Centre for Interdisciplinary Research in Basic Sciences, Jamia Millia Islamia, New Delhi, India

**Keywords:** Mathematical Modeling, Vector-borne disease, SIR model, Basic reproduction number

## Abstract

**Background:**

Viral diseases are highly widespread infections caused by viruses. These viruses are passing from one human to other humans through a certain medium. The medium might be mosquito, animal, reservoir and food, etc. Here, the population of both human and mosquito vectors are important.

**Main body of the abstract:**

The main objectives are here to introduce the historical perspective of mathematical modeling, enable the mathematical modeler to understand the basic mathematical theory behind this and present a systematic review on mathematical modeling for four vector-borne viral diseases using the deterministic approach. Furthermore, we also introduced other mathematical techniques to deal with vector-borne diseases. Mathematical models could help forecast the infectious population of humans and vectors during the outbreak.

**Short conclusion:**

This study will be helpful for mathematical modelers in vector-borne diseases and ready-made material in the review for future advancement in the subject. This study will not only benefit vector-borne conditions but will enable ideas for other illnesses.

## Background

New types of viral diseases are arising day by day. Viral diseases are prevalent infections caused by viruses (microorganisms) worldwide. There are many types of viruses that lead to a variety of viral infections. A few of them are widespread viral diseases, such as COVID-19, dengue fever, chickenpox, influenza, HIV/AIDS, measles, and rubella. Viral diseases are contagious and pass from one individual to another individual through a certain medium. There are several ways to transmit viral infection from one to another. Few viral diseases are transmitted by vectors known as vector-borne viral infections. Vector-borne diseases are human diseases caused by parasites, viruses, and bacteria that are transmitted through the vector. Every year, more than 700,000 people die from diseases such as malaria, dengue fever, Schistosomiasis, African trypanosomiasis, leishmaniasis, Chagas disease, yellow fever, Japanese encephalitis, and onchocerciasis. The burden of these diseases is highest in the tropics and subtropics and affects the poorest disproportionately. Since 2014, dengue, malaria, chikungunya, yellow fever, and Zika have caused population declines, deaths, and overwhelming health care systems in many countries. Other illnesses, such as chikungunya fever, leishmaniasis, and lymphatic filariasis, cause chronic distress, lifelong morbidity, disability, and occasional stigma. Various types of vectors depend upon their transmission method. Some vectors are blood-sucking insects that become infected by disease-responsible microorganisms during the blood-sucking process from the infected host and transmit it into a new normal host during the next blood-sucking process. Mosquitoes are well-known vectors for various infectious diseases. Mosquitoes are responsible for the transmission from one individual to another of serious diseases such as chikungunya, dengue fever, zika fever, yellow fever, West-Nile fever, and Rift Valley Fever (RVF) . Other than mosquitoes, there are other vectors for several diseases; for example, fleas are responsible for plague in which fleas transmitted from rat to human, freshwater snails are responsible for Schistosomiasis, ticks are responsible for Lyme and Tularemia, bugs are responsible for Chagas disease, and there are many other types of vectors [1]. These viral diseases occur mainly in regions where the climate is tropical and sub-tropical, which are suitable for the production of mosquitoes. These diseases are also found normally in areas where drinking water is harmful and the sanitation system is unsuitable. Although, these diseases are not limited to any specific region due to globalization of travelling, and climate change [1]. Antibiotics are ineffective for viral infection in contrast to bacterial infection in humans. However, the viral infection causes illness for a limited period, and then it resolves as the body’s immune system, which enables it to attack the viruses and recover from that infection. In some other cases, the infection may be dangerous and life-threatening. Some epidemics of infectious diseases occurred in $20{th}$ and $21{th}$ century are shown in Tables 1 and 2, respectively. Due to these epidemic/pandemic events, humankind faced a great loss in health and wealth.

**Table 1:** Few major epidemic/pandemic of viral infections in $20{th}$ century

Viral infection	Duration	Location	Death	References
Plague	1910-1912	China	40,000	[2]
Influenza	1918-1920	World-wide	75,000,000	[3]
Influenza (Asian flu)	1957-1958	World-wide	2,000,000	[4]
Small pox	1974	India	15,000	[5]
Plague	1994	India	52	[2]

**Table 2:** Few major epidemic/pandemic of viral infections in $21{st}$ century

Viral infection	Duration	Location	Death	References
SARS	2002-2003	Asia, Canada	775	[6]
Dengue fever	2005	Singapore	19	[7]
Dengue fever	2006	India	50+	[8]
Influenza	2009	World-wide	$\approx$15,000	[9]
Ebola virus	2013-16	West-Africa	$\approx$12,000	[10]
Chikungunya	2013-15	America	$\approx$200	[11]
Zika virus	2015-2017	World-wide	–	[11]
COVID-19	2019-2022	World-wide	6,289,371 (till 01-06-2022)	[12]

These vector-borne viral infections propagate from one individual to another. If we want to do a non-pharmaceutical intervention (such as quarantine, isolation, social distancing and hygiene behaviors, social, physical, psychological, capacity, motivation, economic and demographic that impact on engagement) during the progression of disease in the population. Non-pharmaceutical intervention is due to the treatment of various viral diseases that have been still unknown. Then we must know the factors of propagation and dynamics of the infected population. Thus, mathematical modeling is a perfect tool to deal with this problem. Although it is very challenging to convert the real problem into a mathematical model in an ideal way, it may be constructed under certain constraints. The prediction using a mathematical model will be helpful for the state to formulate policies and control over spreading of disease. The mathematical model formulation process clarifies the infection’s assumptions, variables and parameters. Further, the model also provides conceptual results such as thresholds, basic reproduction numbers, contact numbers, and replacement numbers for the transmission dynamics of diseases. Mathematical models can be utilized as useful experimental tools in understanding the transmission characteristics of infection in communities, regions, and countries that can lead to the formulation of appropriate control measures [13, 14]. In many countries, mathematical models are helpful in decision-making policies about epidemics/pandemics. Mathematical modeling is also a decision-making tool for intervention programs for infectious diseases. In recent times, the mathematical modeling of such viral infectious has many approaches, such as deterministic, stochastic, network-based, simulation, and statistical. The selection of these techniques in a mathematical model depends on the characteristics of the diseases for which model is being created and for the model. In this chapter, we will focus on the deterministic approach only.

To date, many mathematical models have been developed, and many models are underway, such as [15–19]. Although, several models have high significance for the society and government for policy making. During any epidemic or pandemic, we did not spend time reviewing all articles and identifying which model had the restriction and criteria and which model was better or worse as per the situation. Hence, a notion came to mind that it would be good to have a systematic review of the mathematical model of the viral disease. We have picked here four vector-borne diseases: chikungunya, zika, dengue, and west-Nile infection. As we know that mathematical modeling of infectious disease is somehow similar strategies to develop a deterministic model; however, the parameter and variable lying differential. In this survey article, we try to incorporate how one model variable and parameter are different from others and what is novel development and results in the existing surveyed model.

Thus, the organization of the survey follows historical perspectives of mathematical modeling and a systematic review of mathematical modeling on four vector-borne diseases (Dengue, Zika, Chikungunya, West-Nile) in Section 2.1 and Section 2.2, respectively. After that, Section 2.3 elaborates on basic numerical techniques for solving the SIR model and herd immunity and vaccination incorporation concept in the mathematical model. In the last Section 2.4 described different techniques of mathematical modeling apart from deterministic. This survey will be a highly useful mathematical modeler in vector-borne diseases. This survey is not only limited to use for a vector-borne oriented researcher; however, this will also be useful for all mathematical developers, especially newly emerged viral infections.

## Main text

### Historical perspective of modeling for infectious diseases

The origin of mathematical modeling for vector-borne viral infection progression in humans is the modeling of infectious diseases. Therefore, a historical perspective is needed to understand compartmental modeling for formulating the vector-borne infection model. In this section, we have described a historical perspective of mathematical modeling for infectious diseases. Smallpox is a disease which emerged in the period before written records. By the end of the medieval period, this disease had become the main disease responsible for high child mortality in Asia and Europe. Although, the etiology of this disease could not be illuminated till $20{th}$ century. It was the first disease for which a specific intervention, immunization or vaccination was available in the history of infectious diseases. The mathematician Daniel Bernoulli is known for the first mathematical Model for "small pox." He submitted his work entitled *An attempt at a new analysis of the mortality caused by smallpox and the advantages of inoculation to prevent it* to the Academy of Sciences in Paris in 1760. He thought inoculation problem in a mathematical way for smallpox. The task was to compare the effect of inoculation with the normal conditions of infections. For this, he made some assumptions under which he developed the following equation using some basic calculus:

1 $$\begin{aligned} \frac{1}{P} \frac{dS}{dP}- \frac{S}{P^2}\frac{dP}{dx}=-q \frac{S}{P}+pq\left( \frac{S}{P}\right) ^2, \end{aligned}$$

where *S*(*x*) :  Number of susceptible people at age *x* without ever infected by small-pox (independent of age), *P*(*x*) :  Total number of alive people at age *x*, $(1-p):$ survival probability, *q* :  infection probability per year per human. If $\frac{S(x)}{P(x)}=f(x)$ then Eq. 1) can be written as:

2 $$\begin{aligned} \frac{df}{dx}=-qf+pqf^2. \end{aligned}$$

The solution of the similar Eq. (2) was known (given by Daniel’s uncle Jackob Bernoulli) several decades before. The final solution of Eq. (2) was given in the form $f(x)=\frac{1}{p+(1-p)e^{qx}}$. On the basis of this solution he computed life table at different ages *x*. The number of deaths between age *x* and $x+1$ was computed by $pq \int _{x}^{x+1} S(t) dt$ which is equal to $pq \frac{\left[ S(x)+S(x+1) \right] }{2}$ by Trapezoid approximation.

The modern approach for the mathematical epidemiological models was established by the P.D. En’ko between 1873 to 1894. P.D. En’ko presented his work [20] in the form of a mathematical model in 1889 in Russian in St. Petersburg. P.D. En’ko set some specific assumptions known as “first principles”. Based on “first principles”, he developed the iterative method, which is shown in the following system of iterative equations:

3 $$\begin{aligned} C_{t+1}= & {} S_t\{1-\left( 1-\frac{C_t}{N_t-1}\right) ^{kN_t}\}, \end{aligned}$$

4 $$\begin{aligned} S_{t+1}= & {} S_t\left( 1-\frac{C_t}{N_t-1}\right) ^{kN_t}, \end{aligned}$$

5 $$\begin{aligned} N_{t+1}= & {} N_t-C_t, \end{aligned}$$

where $C_t$ is the number of infected individuals, $S_t$ is the number of susceptible and $N_t$ is the total number of individuals during the time interval *t*, *k* is the parameter which determines the number of contacts of a susceptible [20]. In the model, the expression $\frac{C_t}{N_t-1}$ represents the probability that a susceptible person comes in contact with an infected person. The model had two basic characteristics (i) discreteness of time (ii) deterministic nature. He fitted his Model with the real data of measles and scarlet fever outbreaks in two boarding schools, namely the **Imperial Education College for the Daughters of Nobility** and the **Alexander Institute** in St. Petersburg during 1875-1888.

Another work in the history of mathematical modeling is done by British scientist Sir Ronald Ross who discussed the transmission of malaria between mosquito and human, and got the second *noble prize* in the medicine in 1902 [21]. Ross claimed that malaria can be prevented by simply reducing the number of mosquitoes in his book “The prevention on Malaria” published in 1911. He presented a mathematical model for his claim. His mathematical model was based on the system of two ordinary differential equations (ODE) in the following manner:

6 $$\begin{aligned} \frac{dI}{dt}=\, & {} bp'i\frac{N-I}{N}-aI, \end{aligned}$$

7 $$\begin{aligned} \frac{di}{dt}=\, & {} bp(n-i)\frac{I}{N}-mI, \end{aligned}$$

where *N* :  total human population, *I*(*t*) :  number of infected human at time *t*, *n* :  total mosquito population, *i*(*t*) :  number of infected mosquitoes at time *t*, *b* :  biting rate of mosquito, *p* :  transmission probability of infection from human to mosquito per unit bite, $p':$ transmission probability of infection from human to mosquito per unit bite, *a* :  recovery rate of human from infection, and *m* :  mortality rate of mosquito. The terms $bp'i\frac{N-I}{N} dt$ and $-aI dt$ represents the number of new infected humans and number of recovered humans in small time period *dt*, respectively. Similarly, the terms $bp(n-i)\frac{I}{N} dt$ and $-mIdt$ denote the number of new infected mosquitoes and number of mosquitoes that die (assuming infection does not affect the mortality rate of mosquitoes) during the small time period *dt*, respectively. Ross discussed two steady state points $(\frac{dI}{dt}=0$ and $\frac{di}{dt}=0)$ for his model:

1. $I=0$, $i=0$; which always shows the absence of malaria.
2. $$I=N \frac{1-amN/(b^2pp'n)}{1+aN/(bp'n)}$$, $$i=n \frac{1-amN/(b^2pp'n)}{1+m/(bp)}$$.

Ross noticed that for $I>0$ and $i>0$ the number of mosquitoes (*n*) should be greater than a threshold value $n^*=\frac{amN}{b^2pp'}$.

The concrete work on the mathematical modeling for epidemiology was carried out by Sir Ross, R.A., W.H., W.H. Hammer, A.G. Mckendrick and W.O. Kermak during 1900-1935 [22–24]. The work done by these scientists was based on the new approach known as compartment modeling whereas J. Brownlee did the work from a statistical perspective.

The development of compartment model theory is the consequence of the three papers written by W.O. Kermack and A.G. Mckendrick in 1927, 1932, and 1933 [22–24]. The well known and simple model for the transmission of infectious disease is the Susceptible-Infected-Recovered (SIR) model given by Kermack and Mckendrick [24]. In basic SIR model whole population is categorized into susceptible (*S*), infected (*I*), and recovered (*R*) with respect to the time *t* (normally in days). The following ordinary differential equations can represent the transmission of disease:

8a $$\begin{aligned} \frac{dS}{dt}&= -\beta S I , \end{aligned}$$

8b $$\begin{aligned} \frac{dI}{dt}&= \beta S I - \gamma I , \end{aligned}$$

8c $$\begin{aligned} \frac{dR}{dt}&= \gamma I , \end{aligned}$$

where $\frac{dS}{dt}$, $\frac{dI}{dt}$, $\frac{dR}{dt}$ are the rates of change in the quantities *S*(*t*), *I*(*t*), *R*(*t*), respectively, and $\beta$ is the transmission rate which is equal to the average number of infected individuals infected by an infectious individual, assuming that all contacts are susceptible to that individual. $\gamma$ is the rate at which infected individuals recover. Therefore $1/\gamma$ is the average time an infected individual remains infectious. The product $\beta S(t) I(t)$ is the "total infection rate," which reflects the infected population fraction per unit time. The quantity $\beta / \gamma$ is the average number of individuals infected by a single infectious individual treating the whole Population as susceptible. This fraction $(\beta /\gamma )$ is known as basic reproduction number $(R_0)$ and is an important index for invasion of the diseases in the given Population. Hethcote [25] defined the effective reproduction number (replacement number) as:

9 $$\begin{aligned} {\mathcal {R}}= \frac{\beta }{\gamma } S(0), \end{aligned}$$

which provides the average number of secondary infections produced by the single infected individual during the infection. Further, there are two main key conclusions about disease transmission:

1. *The threshold value:* (*a*) If $S(0) R_0 <1$ then disease rapidly dies-out, i.e., there is no epidemic. (*b*) If $S(0) R_0 >1$, the number of individuals will increase rapidly, i.e., epidemic will occur despite of small susceptible portion.
2. *The size:* when an epidemic occurs, it does not depend on the initial number of infected individuals but only depends on the initial Population of susceptible *S*(0) and the basic reproduction number $(R_0)$.

The basic (SIR) model is far from the reality due to the following assumptions:

- *Well mixed and Homogeneously distributed Population :* During the construction of Model, it was assumed that the Population is well-mixed, i.e., every individual has the same probability of coming in contact with any other individual. This hypothesis relaxes many factors, like that contact depends upon the geographical or social relationship. This is also implied that the Population is treated as homogeneously distributed, *i.e.,* each individual has an equal probability of transmitting the infection. This assumption ensures that the Model does not consider the level of susceptibility or infectiousness.
- *Infection duration is exponentially distributive:* Model assumes that an infected individual becomes infectious immediately and recovery per unit time of an individual is independent from the time that has passed since infection. Both these hypotheses are not realistic [26].
- *Large-size population:* During the construction of Model, it is assumed that the Population is very large (usually infinite). For a small population (for a small geographical region), another approach, such as stochastic, is more appropriate [27].

Kermack and Mckendrick also discussed the role of lapsed time $(\tau )$ since infection started in their model. Therefore the transmission of infection $\beta (\tau )$ and recovery from infection $\gamma (\tau )$ depend on the lapsed time $\tau$. In this case reproduction number is calculated as: $R_0=N\int _{0}^{\infty }\beta (\tau )e^{-\int _{0}^{\tau }\gamma (x)dx}d\tau$ (where *N*=total human population).

#### Final size of epidemic

It can be shown that $\lim \limits _{t\rightarrow \infty } {I(t)=I(\infty )=0}$ but $\lim \limits _{t\rightarrow \infty } {S(t)=S(\infty )}$ and $\lim \limits _{t\rightarrow \infty } {R(t)=R(\infty )}$ are finite but their values depend upon the initial conditions. Final size of the total number of cases of the disease outbreak can be determined by the initial conditions and parameters $\beta$ and $\gamma$. If $R(0)=0$ then $N(t)=S(t)+I(t)+R(t)$ implies that $R(\infty )$ will represent the final size of disease outbreak. The value of $R(\infty )$ could be determined by the two Eqs. (8a & 8b) such that 8b is divided by 8a follows as:

10 $$\begin{aligned} \frac{dI}{dS}=\frac{dI/dt}{dS/dt}=\frac{\beta S I-\gamma I}{-\beta S I}. \end{aligned}$$

We get after simplification of Eq. (10):

11 $$\begin{aligned} \frac{dI}{dS}=-1+\frac{\gamma }{\beta S}. \end{aligned}$$

Let us Integrating both sides Eq. (11) with respect to *S*, then we get:

12 $$\begin{aligned} I=-S+ \frac{\gamma }{\beta } \ln (S) +C, \end{aligned}$$

where C is an arbitrary constant of integration. Let initial conditions are $I(0)=1$ and $R(0)=0$ then $S(0)=N-1$. Using the initial conditions the value of *C* can be determined as $N-\frac{\gamma }{\beta } \ln (N-1)$. Therefore, we get the following expression for *I*(*t*):

13 $$\begin{aligned} I(t)=-S(t) + \frac{\gamma }{\beta } \ln (S)+N-\frac{\gamma }{\beta } \ln (N-1). \end{aligned}$$

As $t\rightarrow \infty$ and using $I(\infty )=0$ yields:

14 $$\begin{aligned} S(\infty ) = \frac{\gamma }{\beta } \ln \left[ \frac{S(\infty )}{N-1}\right] +N. \end{aligned}$$

Equation (14) provides the implicit solution for $S(\infty )$. Thus, we can determine the final size of outbreak $R(\infty )=N-S(\infty )$. For example, if $S(0)=99, I(0)=1, R(0)=0$ then $N=100$ and let $\beta = 0.02, \gamma =1$. Then $S(\infty )\approx 19.9$ therefore final size $R(\infty )=80.1$ for given parameter values and initial conditions.

### Systematic review for four different vector-borne diseases

There are various techniques available to convert real problems into mathematical models. The compartmental mathematical modeling approach has novelty in all these techniques used to analyze the transmission characteristics of infectious diseases. In this approach whole population of individuals is divided into different classes based on the epidemiological state of individuals. The letters *S*, *E*, *I*, *R* (*S*-Susceptible, *E*-Exposed, *I*-Infected, *R*-Recovered) are normally used to represent different epidemiological classes in the compartment mathematical model. The selection of compartments in a mathematical model depends on the characteristics of the diseases for which the model is being created. Some classes may be omitted from a model if they do not play a crucial role in the model. According to the presence of compartments, acronyms for different mathematical models can be used as SEIRS, SEIR, SIRS, SIR, SEIS, SEI, SIS, SI, etc.

Except above historical development of mathematical modeling, many researchers proposed their models with some more assumptions and restrictions to achieve the goal of continuous time intervals and provided the solution to real problems more accurately [28, 28, 29]. In the continuation of this series, some are described here. Hetcote [13] introduced the basic assumptions, notations, concepts and derivations of formulas for the epidemiological models. Classification of infectious diseases is done based on agent and mode of transmission, which is similar to the classification given by H. Dietz in 1974. He discussed three basic epidemiological models namely (SIS) model, (SIR) model without vital dynamics and (SIR) model with vital dynamics. He also talked about herd immunity and the vaccination concept. Hetcote and Ark [30] gave the model for heterogeneous population. They found contact matrices on the assumption of proportionate mixing. They also estimated the parameter for the homogeneous and heterogeneous populations. They analyzed this model for three immunization programs and compared them. Fraser et al. [31] discussed two main factors in controlling the outbreak of an infectious disease in the absence of vaccination (i) isolation of symptomatic individuals (ii) contact tracing and quarantining of symptomatic individuals. They claim that these measures depend upon the symptoms of specific diseases. Therefore the relative time for infectiousness and visibility of symptoms cab be estimated by the proposed mathematical model. Fan, Li and Wang [32] talked about the global stability of a (SEIS) model with constant recruitment and varying total population. They showed that global stability is fully governed by $R_0$ in such a way that if $R_0\le 1$, then stability is global, and disease will die out, but if $R_0 >1$, then a unique endemic equilibrium exists. McCallum et al. [33] discussed the facts about modeling pathogen transmission. They talked about mass-action assumptions and pointed out that models based on mass action have often been modeled incorrectly. Yusuf *et al.* developed a model for HIV using fractional order-based differential equation and logistic growth model, these fractional based techniques are emerging nowadays and provide us best fitted with actual outbreak data [34–40]. In their opinion, alternative models of transmission are available for better results.

In the past few years, viral diseases like Zika, dengue, chikungunya, West Nile and yellow fever have gained considerable attention. The threat of these viral infections is a broad area of concern. The ecological assessment of the pandemic threat of Zika virus (ZIKV) has been given by C. Carlson [41]. In 2010, the continuous global threat of dengue was presented in [42], concluding that the control measures like antiviral vaccines and drugs should be developed, which will make an important contribution to the upcoming dengue outbreak. In this respect, Mathematical modeling arises as a powerful tool to investigate the transmission and interior of various viral diseases to develop control measures [25, 43–55]. These are basically compartmental models which divides the population into different compartments comprising of different infectious diseases. Different mathematical models have been proposed with some more assumptions and restrictions to achieve a goal by providing more accurate solutions to real problems. Here, some models have been described with more assumptions and solutions for different viral diseases.

#### Mathematical modeling for dengue fever

Dengue infection is caused by the dengue virus (DENV), a mosquito-borne flavivirus. The symptoms of dengue infection are headache, vomiting, joint pains, high fever, skin rash, etc. These symptoms usually start from 3 to 14 days after getting the infection. The recovery time from DENV ranges from 2 to 7 days. The severe complications due to DENV infection may be low blood platelets and blood plasma levels, low blood pressure, etc. A. Aegypti female mosquitoes are responsible for transmitting DENV infection from one individual to another through their bites. There are different serotypes of the DENV. Recently, a vaccine for DENV infection has been approved and is available in some countries. However, it is only recommended for individuals who are not infected previously. Dengue infection has become a global issue since the second world war. Nowadays, it is spread in more than 110 countries, mainly in Asia and South America. It has been proven as the most increasing infection as the number of cases has increased 30 times in the last 50 years. The disease transmission has its highest rate in the season of rainfall and warmer temperatures [12].

Previously, several approaches have been used to investigate transmission dynamics of the Dengue virus (DENV) infection, but the mathematical approach is found to be more significant as the epidemiology of DENV infection comprises ecological characteristics and epidemiological determinants. An exhausting literature review is available to discuss, but only a few are being described here. Andraud et al. [56] reviewed the epidemiological models for dengue virus infection using a systematic structural approach. They searched the articles up to 2012 from standard databases, such as Pubmed and ISI Web of knowledge. They found 655 peer-reviewed articles related to *Dengue Epidemic model* after a systematic search. They excluded 16 non-English articles and then 389 duplicate articles. Thus, there were only 373 articles for screening. Three hundred thirty-one articles were found irrelevant out of 373 articles. So finally, 42 articles were finalized for qualitative analysis. In this series, Dietz [57] was the first who propose a deterministic compartmental model for DENV with vaccination presenting critical population size, analytic solution, spatial heterogeneity, age structure and vaccination strategy with the highest basic reproduction number $(R_0)$ equal to 27. Another model in 1992 was developed by Newton et al. [58] to evaluate the impact of Ultra-Low Volume(ULV) Insecticide on dengue epidemics finding that it has little impact on disease transmission. The dynamics of the DENV transmission were done using the SEIR-type model, including the incubation period for both the host and vector population. The model’s basic reproduction number, $R_0$ set, was 1.9, which predicted an asymptotically stable endemic state of model reproducing epidemic transmission. Hence, decreasing the favorable environmental conditions for mosquitoes and $R_0$ is very efficient in reducing virus transmission. A complete vector-host dynamics in a multiple strain epidemiological system was developed in 1997 by Zhilan Feng [59]. Instead of including any exposed compartment, the model included the existence of a second co-circulating strain which causes secondary infections in susceptible or recovered individuals. The analysis shows the existence of an unstable endemic state which provides an environment where dengue serotypes co-circulate, resulting in the competitive exclusion of one strain. Hence, concluding that the existence of competitive exclusion in the model comprises host superinfection process and frequency-dependent contact rates.

Next, the global stability of endemic equilibrium was concluded as one of the parameters in DENV dynamics given by Esteva [60] using a vector-host compartmental model. Various improvements in this model suggested that three threshold parameters exist which regulate the endemic equilibrium [61, 62]. In 1994, a simplified deterministic compartmental model [63] was designed to estimate DENV transmission and concluded that the dengue virus is the only moderately infectious in Brazil. With the increased dengue virus risk, researchers and mathematicians developed different models, including various parameters, to eliminate the disease. In 2003, a simple deterministic model was designed to determine the parameters to develop a dengue transmission model for Brazilian cities and analyzed how control measures are influenced by vector density spatial heterogeneity [64]. The model was parameterized using probability density functions in a range of probable values for each parameter and consists of an expression for basic reproduction number $R_0$ that incorporates vector density spatial heterogeneity. Hence, concluded that the main entomological parameters worth estimating in the model were mortality rate and the extrinsic incubation period, even in the presence of vector density spatial heterogeneity. In the end, suggesting that better approaches to measuring transmission dynamics should be made together by mathematicians, epidemiologists and entomologists to decrease the risk of a DENV infection invasion in Rio de Janeiro.

The Dengue virus is found with four serotypes, DEN-1, DEN-2, DEN-3 and DEN-4, and the interaction of any of the four serotypes with susceptible humans causes dengue. *Aedes aegypti* and *Aedes albopictus* are the two mosquitoes that are recognized dengue causing viruses. Dengue epidemic caused by only one virus or two viruses acting simultaneously [59] were considered using different SEIRS models [58] and SIR models [60]. Therefore, a model is proposed where two different viruses acted on separate time intervals [65]-the paper deals with a succession of two dengue epidemics resultant of these two different viruses. In the proposed model, simulation for the different values of parameters was carried out, giving stability to equilibrium points. Overall the model helps to understand that prevention measures such as chemical methods or vector control through environmental management are insufficient to prevent the disease dynamics as they only help delay outbreaks. It was found that the vaccination is only applicable for the short term as it works against the four serotypes simultaneously. Hence, researchers can investigate and concentrate on the search for a vaccine that focuses on each serotype instead of looking for a vaccine that targets the four serotypes simultaneously.

Since the period of 1950s, Bangkok was highly affected by four DENV serotypes, all of which had co-circulated occurring epidemic outbreaks every ten years [66]. Seeking through the prevention of outbreak occurred, it was noticed that productive management of DENV can be possible by understanding the factors that drive epidemiological patterns. The epidemiological theory with phylogeny data analysis contributes positively to understanding the infectious diseases at intra- and inter-host levels [67]. B. Adams [68] used such an approach concluding that epidemic patterns in Bangkok may be the result of cross-protective immunity between serotypes. The phylogeny analysis of distinct epidemic patterns between four serotypes results that there is an immunological reaction between the serotypes. Hence, the deterministic mathematical model was used to vary these interserotypic immune reactions, which concluded that moderate cross-protective immunity account for the alternating epidemic pattern of DENV circulating in Bangkok. Dengue Hemorrhagic Fever (DHF) concerning Dengue Fever is significantly affecting tropical and subtropical areas across the world. In 2006, an SIR model of DENV transmission was designed with a severe DHF compartment aiming to find the control measures to decrease the number of DHF patients. Analyzing this model reports that there are four equilibrium points, one the disease-free, and the other three correspond to the presence of a single serotype and the coexistence of two serotypes. A large number of average-age DHF cases were also reported in Thailand without knowing the cause. With the decrease in the mosquito population and declining contact between humans and mosquitoes, it is found that demographic transition reduced dengue transmission and also increased the interval between large epidemics [69]. An age-specific deterministic model was designed which examine the impact of secular demographic transition. It is found that a reduction in mortality increases the longevity of immune individuals, which finally decreases the risk of DENV infection in susceptible individuals. The reduction in the birth rate decreases a large number of susceptible individuals, reducing the risk of infection. Hence concluding that demographic transition has a broad impact on dengue transmission dynamics in Thailand and many more countries in Southeast Asia [70–72]. Vertical transmission in mosquitoes has a role in the persistence of dengue as described in [73]. Using a deterministic model, they concluded that vertical transmission at low infection rates is not a key factor for long-term DENV persistence. They also concluded that vertical transmission had no major effect on DENV persistence. In 2011, Johansson reviewed the different mathematical approaches to study dengue transmission dynamics, focusing on estimation methods for basic reproduction number and their consequence on the impact of vaccination [74]. Another review done in 2012 by Andraud et al. [56] includes all the research articles on deterministic models of DENV transmission in humans, linking the model structures with the assumptions based on entomological and epidemiological studies. The key finding of this review mentions that the inclusion of vector components in a four-serotypes model is very important to find the best combination between vector-control measures and vaccination strategies in dengue-prone areas.

One more recent compartmental model was given by Guanghu Zhu [75] mentioning the effects of temperature, mosquito control and human mobility on DENV transmission in the 2014 dengue outbreak in the Pearl River Delta (PRD) in China. These multi-scale factors were introduced to clarify DENV spatiotemporal transmission’s hidden dynamics and capture its internal mechanism. The results show that 1) Human mobility is one of the factors which leads to disease spread across different cities, 2) The suitable temperature conditions were responsible for a disease outbreak in the PRD and 3) The mosquito control measures have a significant role in dengue reduction. This study also reflects that modeling for dengue in population dynamics is one kind of set example for many model deployments in the field of vector-borne viral infection modeling.

#### Mathematical modeling for Zika virus

Zika virus (ZIKV) infection is emerging disease and it is known since 1952 and was originally identified in Africa [76]. The first largely ZIKV infected human reported as an outbreak in Yap, Micronesia during April - July, 2007 [77], followed by an outbreak in French Polynesia between October 2013 and April 2014 [78], and cases in other Pacific countries. Another outbreak occurred in South America in 2015 due to quick mutation in ZIKV [79]. *Aedes aegypti* is known as main carrier in the human population for ZIKV infection. Therefore, ZIKV is likely to be capable of sustained transmission in other tropical areas. There are several other routes that has been observed such as sexual contact, blood transmission, and mother to her fetus. In case of mosquito as vector, the transmission is bidirectional, *i.e.*, an infected mosquito can infect the healthy human and a mosquito may be infected by an infected mosquito. Symptoms of ZIKV infection are headache, joint pain, arthritis, skin rash, nausea, wild fever, etc. Some serious complications due to this infection has been identified as neurological disorder in new born babies (Microcephaly) and in adults (GB syndrome). Additionally, this infection may be responsible for severe thrombolytic, miscarriage, still birth, etc. There is no vaccine is available against Zika parthenogenesis. Therefore prevention plays the crucial role in the progression dynamics of this infection.

It is interesting to draw an attention to mathematician that to quantify the factors of zika infection and prediction at different conditions. Dynamics of infection during 2013-14 Zika outbreak in French Polynesia was analyzed using a mathematical model [80] and a fractional order network model for Zika is also studied in [81]. In-spite of this a lot more admirable studies has been done contributing to the Zika virus analysis [82]. Many researchers have introduced deterministic models for ZIKV that take into account the transmission by vector species only. There are some evidences that sexual transmission is also responsible for disease transmission [83]. But this transmission process was not given that importance as very small number of cases were confirmed in comparison to that of mosquito bites. In 2015, the major transmission by mosquito bites was reported in South America since 1950, due to the favoring temperature conditions. Climate conditions like temperature and rainfall seem to be responsible for the highest biting rate increasing the growth of mosquitoes. In 2015, over South American continents, the warm climate in association with El-Nino exceptionally contribute in ZIKV transmission. Therefore, in 2016 Caminade, Cyril, et al. [84] developed a global mathematical model considering vectors only for transmission risk of ZIKV revealing the role of El-Nino 2015. Further, Tunner et al. [85] extended the previously developed two-vector mathematical framework for an animal vector borne disease (VBD) to ZIKV. The extended model includes one host: human and two vector species: *Aedes aegypti* and *Aedes albopictus*, where former is highly active transmitter of human virus and the later one is less frequent in transmitting and acquiring human virus [86, 87]. The result confirmed that *Aedes aegypti* is a larger threat than *Aedes albopictus* worldwide for ZIKV transmission. But, the threat caused by *Aedes albopictus* should not be ignored especially during the warm climate conditions in temperate regions. Also, the seasonal estimates of basic reproduction number $R_0$ was derived using historical climate data for the period 1950-2015 as it seems to be highly sensitive to climate conditions [88]. Hence, The results found that the overlapping of both the vector species produces the highest $R_0$ value.

In an another interesting study, Gao, Daozhou, et al. [89] designed a mathematical model to study the effect of sexual transmission and mosquito-borne on the spread, control and prevention of ZIKV. As many cases examined for ZIKV, it was found that virus is transmitted in humans by the bites of Aedes mosquitoes. Few of these cases indicates that ZIKV can also be transmitted through sexual contact of humans. Hence, a deterministic model of Zika disease transmission was introduced that consider both mosquito-borne and sexual transmission mode for the reported cases in Brazil, Colombia and El Salvador. To understand the transmission mechanism, SEIR-type model for human was used which is divided into six classes: Susceptible, Exposed, Symptomatically infected, Convalescent, Asymptomatic-ally infected and Recovered and SEI-type model for mosquito which is divided into three classes: Susceptible, Exposed and Infectious. Common parameter values were shared by three of the countries except the initial conditions and population size. Three additional assumptions were made for the proposed mathematical model as follows:

- ZIKV infected humans can not infect mosquitoes asymptomatic-ally
- The sexual ratio is 1:1 for male and female assumed to be subjected to almost same epidemiological factors
- The end of viremic period accompany the escape of symptoms in symptomatically infected human

In continuation, sexual transmission is not mainly responsible for the epidemic outbreak but only increases the risk of infection in humans. The study indicated that the transmission occurred by sexual activity was less than that of the total transmission contributing a percentage of 3.044. The basic reproduction number $(R_0)$ was estimated 2.055 for given data. The overall analysis of model indicates basic reproduction number $(R_0)$ is more sensitive to the mortality and biting rate of mosquitoes while transmission through sexual contact just add to the risk of infection and epidemic size. Only homogeneous mixing human population was assumed while the heterogeneity such as religion, culture and gender which is most difficult to analyze and formulate should be further investigated.

Further, zika has also been reported to be linked with neural defects and congenital anomalies such as microcephaly. In December 2015, the European Centre for Disease Prevention and Control expressed some possibility that ZIKV, congenital microcephaly and Guillian-Barre are associated with each other [90]. A total 2782 cases of microcephaly were reported in Brazil where-as only 147 cases and 167 cases were reported two year prior to ZIKV introduction [91]. Data from French Poynesia also covered a usually large number of cases where babies were born with neural defects during ZIKV outbreak [92]. Hence, a new deterministic mathematical model was developed which considered human vertical transmission of ZIKV, i.e., new born babies with microcephaly and asymptomatically infected individuals [93]. The model consists of new system of ordinary differential equations where two human population: adults, new born babies and one vector population were considered. Few theoretical findings by the model analysis were:

- The most important parameters for ZIKV spread were found mosquito biting rate, mosquitoes death rate, mosquitoes recruitment rate, adult recovery rate and the transmission probability per contact to adult humans and mosquitoes.
- Personal protections were found more effective in reducing the infectious disease as compared to mosquito-reduction strategy by numerical simulations using mosquito control.
- Combined strategy of personal protection and mosquito control is most effective to prevent ZIKV transmission and reduce microcephaly.
- The rate of infections from asymptomatic individuals to mosquitoes increase with the level of infection.
- The model is globally and locally asymptotically stable when $R_0$ is less than or equal to 1 and unstable when greater than 1.

Many more compartmental mathematical models have been developed to gain understanding of control and transmission of infectious diseases, which can further be used to address serious issues. Different strategies for prevention and control of Zika virus were concluded by Ding et al. in their study [94]. It was accomplished by constructing a compartmental mathematical model, which describes the ZIKV transmission dynamics. The following prevention and control measures were concluded: decreasing contact rate of humans and mosquitoes by using mosquito nets, windows and clothes, increasing the autoimmunity to reduce the transmission rate from mosquitoes to humans, rising the death rate of mosquitoes by using insecticide. As different control parameters were introduced in mathematical models to reduce ZIKV transmission, the impact of media is one of the recently included parameter [95]. The mathematical model formulated by inclusion of impact of media consider SIR-type model for host (human) and SI-type model for vector (mosquito) population by involving the possible routes of ZIKV transmission: mosquito borne and sexual transmission. Computed value of basic reproduction number depicted that the model is locally asymptotically stable when $R_0 < 1$ but occurrence of some backward bifurcation indicated that decreasing the value of $R_0$ below one is not enough to prevent the ZIKV. Therefore, model was further extended by introducing the following two types of control parameters:

1. Usage of mosquito repelling creams, electronic devices and the bed nets to reduce the biting rate of mosquitoes.
2. Usage of effective medicines to treat infectious humans.

The results also depicted that efficacy and importance of the role of media in the proposed epidemic model and concluded that the rate of transmission through sexual contact and mosquito biting rate are the key parameters in transmission dynamics of ZIKV.

In this way, there are several factors quantified by the modeling of ZIKV, the quantification of disease factors were further used as the strategies and enabled to envade the disease at several states of the world.

#### Mathematical modeling for Chikungunya virus

Chikungunya infection is also a viral infection caused by the genus alphavirus. Chikungunya virus (CHIKV) is transmitted by the Aedes aegypti and Aedes albopictus from an infected individual to healthy individual by their bites. Although, *Aedes albopictus* is recognized as the principal vector for virus transmission, and also known as the *Asian tiger* mosquito. The symptoms of CHIKV appear from 4 to 7 days after being bitten by an infected mosquito. The symptoms include headache, muscle pain, high fever, skin rash, fatigue, and joint pain (lower back, ankle, knees, wrist, etc). CHIKV infection shares some symptoms and clinical signs and may be misdiagnosed where dengue is common or diagnosis facility is not proper. This infection may be detected by serological tests. The recovery from infection develops the long-life immunity against the virus. Chikungunya virus was first appeared in Tanzania from 1952 to 1953 epidemic [96] and continued to be active until 1970s, and almost disappeared after that. Africa and Asia are two continents where Chikungunya epidemic occurred frequently. In present time, the number of countries at risk for CHIKV infection is more than 40 in which recent outbreaks have occurred.

To control chikungunya outbreak across the world, mathematical compartmental modeling has become an important tool to study how the vector-borne diseases actually evolve. Upto date, several deterministic models have been designed for Dengue and Zika, but only a limited were proposed for Chikungunya. We will discuss here the different models developed to analyze and control several epidemic outbreak occurred. The study of mathematical modeling of CHIKV infection was started in 1970 [97]. After that, no significant study was done in the field of modeling for this disease epidemic. As the disease re-emerged in 2007, the study of Chikungunya model started again. After 1970s the virus started to re-appear in Thailand in 1988 making it one the country which is highly affected by chikungunya reporting about 400 cases per week at the end of 2008 [98]. In Thailand, as the existence of mosquitoes is mostly dependent on the temperature and the season, P. Pongsumpun [99] formulated and analyzed a compartmental model for Chikungunya by including the effect of season which makes impact on the vector population. Here, the disease transmission in vector-human transmission is studied through dynamical model analysis, concluding that the higher rate of transmission from human to vector leads to the higher individual vector proportions. Hence, the outbreak of disease can be eliminated by reducing the transmission rate of the disease. A prominent attempt for compartmental modeling of chikungunya was done by Nicolas Bacaer concerning fluctuation in vector population for the epidemic outbreak in *La Reunion* in March 2005 [100]. The idea of this temporal model was taken from the first model developed for Malaria by Ronald Ross in 1911 [101]. The study concerned with SEIR type model for human population (constant) and SEI type model for vector population (periodic). The major purpose of including such compartmental models was to give an approximate formula for basic reproduction number $(R_0)$ for the 2005-06 chikungunya epidemic in La Reunion considering vector population as fluctuating. This developed approximation formula can be further used for many other different epidemic models.

Another approximation of $(R_0)$ was presented for the cities of Reunion Island in 2008 by Dumont et al. [102]. Chikungunya virus infection emerged on Reunion Island in 2005, reporting the first case in January 2005, and affecting almost one-third population at the end of May 2006. A new temporal mathematical model was developed to investigate the link between the period of 2005 and the outbreak of 2006. In the model, human population was classified into four compartments: Susceptible ($S_h$), Exposed ($E_h$), Infected ($I_h$), and Recovered ($R_h$) considering human population to be constant and vector population is also classified into four classes: Susceptible ($S_m$), Exposed ($E_m$), Infectious ($I_m$), and Larval ($L_m$). They included Egg, larva, and pupa stages in larval class. They also concluded that for the value of $R_0$ less than 1 there exists a locally asymptotically stable disease-free equilibrium. It was also observed that $(R_0)$ varied from place to place on Reunion Island, suggesting that fast and attentive measures such as destruction of breeding sites would be very efficient in controlling the spread of chikungunya. Further, this model was extended in 2010 by incorporating two new parameters: average lifespan of exposed mosquitoes and average adult lifespan for the infected mosquitoes[8]. The study mainly compares various mosquito control tools, finding that following are some elements contributing to stop the disease spread/transmission with a minimum impact on the environment:

- Life-span of infected mosquitoes;
- The adulticide-mechanical control combination, i.e., combining mechanical control, adulticide and larvicide;
- Starting date and duration of the treatment;
- Mechanical control like destruction of breeding sites;

In addition, this study suggested that immature stages of mosquito population should be split into three differential equations for the eggs, pupae and larvae for better understanding of the epidemic and also mentioned that doing this would make the model complex by creating mathematical difficulty.

The temperate climate country Italy has experienced the first largest CHIKV outbreak in 2007 during summer. A study was done that gives an estimate of transmission potential of CHIKV virus for Italy outbreak using a new model comprising the temporal dynamics of *Aedes albopictus* suggested by Dumont et al. [103]. The model stimulated the vector abundance in four life stages: eggs, larvae, pupae, and female adults. The main aim of modeling the dynamics of the vector is to find an appropriate value of the ratio of vectors to human by giving an estimate of mosquito abundance during the outbreak which is further used to calculate the important parameters and rates of epidemic and for controlling purpose. The results confirm that vector-borne diseases have higher risk in temperate climate countries and the disease spread can be controlled by timely intervention even though the transmission potential of virus is quite high. The first model to consider CHIKV transmission in USA was developed in 2012 [104]. The model comprises of climate-based mosquito population dynamics with an epidemiological model to see the effect of temperature on outbreak risk. The SEIR type model was designed for human population and SEI type model was designed for adult vector population while the immature vector stages are divided into four compartments: eggs, larvae, pupae, and eggs undergoing dispause. The results suggest that the regions where temperature supports mosquito growth have higher risk of outbreak, so such regions should be identified to plan various intervention measures. Results also suggested that decreasing the host exposure to infected mosquitoes and reducing vector population could be very efficient in minimizing the risk of disease outbreak.

A few mathematical models were designed for Chikungunya dynamics and control for Reunion Island epidemic over the period of 2005-2006 [102, 103]. These models neglected some biological components which were further included in a model developed in 2013 [105]. Two new independent influential parameters included in the model were the rate of symptoms arrival and the rate at which humans become infectious. The model mainly described the robust influence of latent period and pre-latent period of infection in human by using sensitivity analysis using Monte Carlo simulation. The result demonstrated that distinguishing these two parameters highly contributed to accurate model fitting and to initially inform to control. A new mathematical deterministic model was developed to study three age-structured transmission dynamics of CHIKV infection [106]. The age groups are divided as: juveniles, adults and seniors. Three different strategies to control and reduce chikungunya cases were also implemented, i.e., personal-protection, mosquito reduction and universal strategy in order to reduce the chikungunya cases. The paper contributed in various theoretical and epidemiological findings mentioned as follows:

- The three age-structure model was both locally and globally stable when the associated basic reproduction number $(R_0)$ is less than 1 and unstable when greater than 1.
- Quantitative dynamics with respect to the local and global stability and backward bifurcation properties were not influenced by inclusion of age structure.
- The sensitivity demonstrate the dominant parameters: death rate of mosquitoes, mosquito recruitment rate, mosquito biting rate, and transmission probability per contact in humans as well as in mosquitoes.
- The dominance and sensitivity of these parameters did not alter by incorporating age structure in chikungunya model.
- It was found by the help of numerical simulations that eliminating age structure shows that the age distribution is not necessary for effective control strategy.
- The numerical simulations also suggested that among the different control strategies the mosquito-reduction strategy is more effective as compared to personal-protection strategy.

Further, the dynamics of CHIKV infection during 2015 outbreak in Colombia have been analyzed through deterministic mathematical modeling [107]. The newly designed model included a chronic sub-population, which was not included in any other previously proposed model. In chronic sub-population, human cannot transmit disease but had some type of chronic rheumatic symptoms. The model was formulated in order to investigate the importance of specific parameters concluding that decreasing the transmission parameters would reduce the diffusion of CHIKV infection. The results showed that reducing the basic reproduction number $R_0$ to less than 1 helps in disease disappearing. Results also concluded that decreasing the number of infected mosquitoes will affect $R_0$, i.e., the value of $R_0$ decreases by increasing the mortality of vector population. Finally, they concluded that the health policy should be implemented to increase the mortality rate to reduce the number of chikungunya cases in considered population. It is also implied that CHIKV outbreak did not occurred from last several years and did not find sufficient literature on mathematical modeling since 2015.

#### Mathematical modeling for West-Nile virus

West-Nile virus (WNV) infection is caused by a mosquito-borne zoonotic arbovirus which is a neuropathogen for humans, horses, birds and belongs to the family Flaviviridae. West-Nile virus (WNV) is named so as it was first isolated from a woman in the West Nile, a district of Uganda in 1937 [108]. WNV mostly infects birds as compared to humans by the bite of female mosquitoes which in turn infected by feeding from the blood of infected birds. WNV transmission via blood transfusion, tissue transplant, and mother-to-child is not reported [109]. Further, humans and other mammals are considered as dead-end host for WNV infection [110, 111]. The main symptoms noted are headache, fever, body aches, nausea, swelling, and inflammation of the spinal cord and in human’s brain [12].

The viremia in infected birds remains for the period from 1 to 7 days depending upon the species of birds. During this time infected birds can transmit the virus to the susceptible mosquitoes and recovered from infection acquiring the life-time immunity against the WNV infection [112]. Birds can be classified into two categories on the basis of duration of viremia: highly competent hosts (HCH) and mildly competent hosts (MCH) [113, 114].

First outbreak of WNV infection was in New York state in 1999 [115, 116]. In 2000, this infection expanded in 12 states of Columbia district [116]. Currently, WNV infection has been exhausted throughout the North America in many avian and mosquito species [117]. Moreover, more than 2.5 million individuals has been infected with WNV infection during 1999–2010 and more than 1300 deaths [118].

There are less studies related to the field of mathematical modeling of the transmission of WNV. As far as we know, Thomas and Urena [119] gave first time a mathematical model based on difference equation to study the transmission dynamics of WNV. The main purpose of the model was to target the effects of WNV on New York City, and in turn calculate the amount of spraying needed to get rid of the virus. Wonham et al. [120] used a single-season ordinary differential equation model for WNV transmission targeting mosquito-bird population which shows that if mosquito control decreases outbreak threshold or WNV, bird control increases. Seeking the above two models, in 2005 a single-season ordinary differential equation model was formulated targeting mosquito-bird-human community for transmission dynamics of WNV [121]. The model examined the temporal dynamics of the mosquito-bird-human population where mosquito population was classified as uninfected female mosquitoes $M_u(t)$, infected female mosquitoes $M_i(t)$, bird population is classified as uninfected birds $B_u(t)$, infected birds $B_i(t)$, and human population is classified as susceptible humans $S(t)$, asymptomatically infected humans $E(t)$, symptomatically infected humans $I(t)$, hospitalized WNV-infected humans $H(t)$ and recovered humans $R(t)$. The main aim of this paper is to use mathematical modeling to analyze the WNV transmission dynamics and to assess two main WNV preventive strategies namely: mosquito reduction strategies and personal protection. Jang et al. [122] proposed their mode for West Nile epidemic model in discrete-time. The model comprised of vector and avian population, where the vector population is classified as susceptible or infective class and the avian population was classified into susceptible, infective, and recovered classes. The study concluded that the disease-free equilibrium is locally asymptotically stable if $(R_0<1)$ and possesses a unique endemic equilibrium if $(R_0>1)$. Hence, they showed that the disease can persist in the populations if $(R_0>1)$. Blayneh et al. [123] revealed the existence of the phenomenon of backward bifurcation in WNV transmission dynamics in their study. In a recent study, Abdelrazec et al. [124] established a compartmental model to study the dynamics of transmission of WNV in mosquito-bird cycle and humans. The model was formulated into three steps. Firstly, model was established and then studied without seasonality and proved the existence of the backward bifurcation of the model. Secondly, the model was extended to include the seasonal variations to examine the effect of seasonal changes on virus transmission. Further, the model was extended to assess the impact of some anti-WNV control measures.

### Numerical solution, herd immunity and vaccination in basic SIR model

**Fig. 1:**
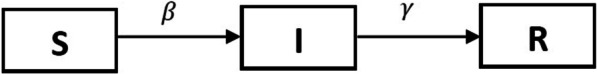
Compartmental structure of basic SIR model

**Fig. 2:**
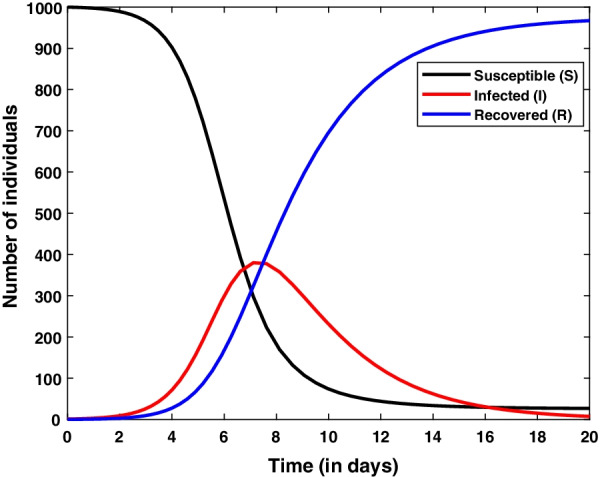
Numerical solution of basic SIR model. Model is simulated for initial values $S=1000, I=1, R=0$ and parameter values $\beta =1.5 \times 10{-3}, \gamma =0.4$

The basic SIR model (As shown in Fig. 1) given by the system of ordinary differential Eqs. (8a–8c) are nonlinear and coupled in nature. Therefore it is not an easy task to solve this model analytically. This issue can be resolved by solving the given system of ordinary differential equations numerically. There are several methods available to solve these equations. For demonstration purpose, we solve given SIR model using *ode45* function in MATLAB which is based on Runga-Kutta method of $4^{th}$ order. To solve given SIR model numerically, we are required parameter values and initial conditions for state variables *S*, *I* and *R*. We have chosen these values randomly and simulated the solution curves which are shown in Fig. 2.

**Fig. 3:**
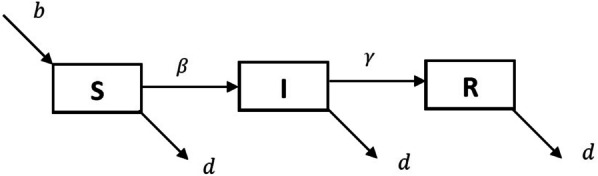
Compartmental structure of SIR model with vital dynamics

**Fig. 4:**
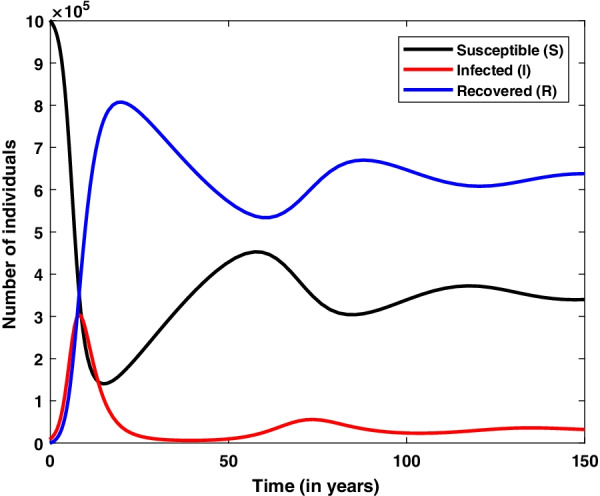
Numerical solution of SIR model with viral dynamics. Model is simulated for the initial values $S=10^6, I=10^4, R=0$ and parameter values $b=1/60, d=1/60, \beta =10^{-6}, \gamma =1/3$

Solution curves depict that susceptible population reduces and recovered population increases with respect to the time. Whereas infected population increase up-to a limit and starts decreasing after attaining a peak. From Fig. 4, it is also depicted that all three population reach at a constant level.

#### Endemic situation

Endemic situation refers the stage in which infection persists in the population at each time interval. In result, the outbreaks of infection occur time to time in the considered population. There are several childhood infections which show this type of characteristics such as measles and chickenpox. This type of model is normally used to study the dynamics of infection at larger population size such as a city or a country. For endemic case we are required to consider the natural birth and death in the basic SIR model. Such model is used to explain endemic situation as shown in Fig. 3 and equivalent mathematical framework is given by the system of Eqs. (15a–15a):

15a $$\begin{aligned} \frac{dS}{dt}&=b N -\beta S I -d S , \end{aligned}$$

15b $$\begin{aligned} \frac{dI}{dt}&= \beta S I - \gamma I -d I , \end{aligned}$$

15c $$\begin{aligned} \frac{dR}{dt}&= \gamma I - d R , \end{aligned}$$

where $\beta$ and $\gamma$ are infection rate and recovery rate, respectively, same as described in basic SIR model (8a-8c). The total population size is given by $N=S+I+R$. The parameters *b* and *d* are natural birth rate per capita and death rate in the given population, respectively. If we add all three Eqs. 15a–15c, we get the following differential equation:

16 $$\begin{aligned} \frac{dN}{dt}=(b-d)N. \end{aligned}$$

**Fig. 5:**
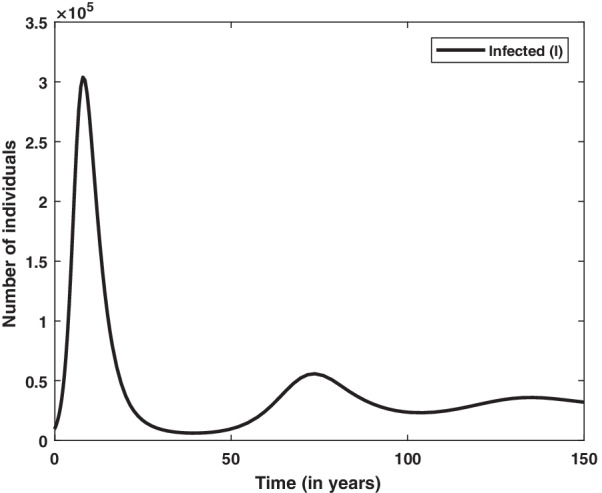
Numerical solution of SIR model with viral dynamics. Model is simulated for the initial values $S=10^6, I=10^4, R=0$ and parameter values $b=1/60, d=1/60, \beta =10^{-6}, \gamma =1/3$

If $b=d$ then $\frac{dN}{dt}=0 \implies N=$ constant. This is the hypothesis under which the total population remains constant over the time. Numerical solution curves for state variable *S*, *I*, *R* are shown in Fig. 4 at choosen values of parameters and initial values of state variables. More specifically, solution curve for infected population (*I*) is shown in Fig. 5. In the initial phase of time it is similar to the basic SIR model but after some time infected individual rebounds instead of dying out. In this case, we get damping oscillations for the infected population and finally it tends to a steady state. Therefore infection always persists in the given population.

**Fig. 6:**
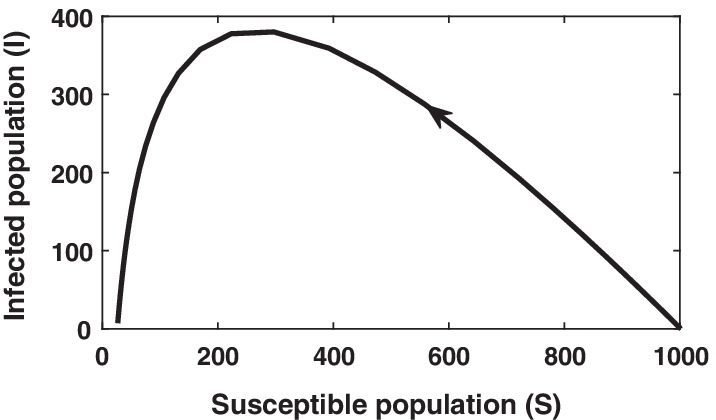
Phase plane analysis of basic SIR model. Simulation is done for initial values $S=1000, I=1, R=0$ and parameter values $\beta =1.5 \times 10{-3}, \gamma =0.4$

**Fig. 7:**
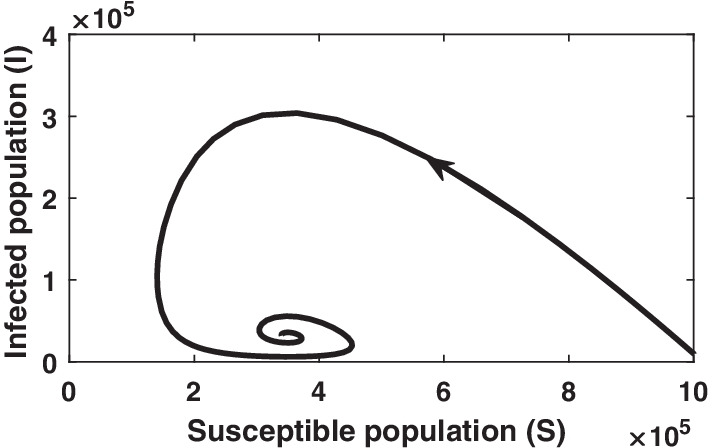
Phase plane analysis of SIR model with vital dynamics (endemic case). Simulation is done for the initial values $S=10^6, I=10^4, R=0$ and parameter values $b=1/60, d=1/60, \beta =10^{-6}, \gamma =1/3$

To know the behavior of solution without solving the given system equilibrium point and phase-plane analysis are two important key points. Phase plane trajectory in $SI-plane$ for basic SIR model and SIR model with vital dynamics are shown in Figs. 6 and 7, respectively. Figure 6 confirm the behavior of infected population with respect time to shown in Fig. 2 as it dies out after reaching at the maximum level. The spiral curve present in Fig. 7 also confirms the damping oscillatory behavior of infected population which is shown in Fig. 5.

#### Herd immunity and vaccination in SIR model

Susceptible population can be reduced through the vaccination process. Although, vaccination to all $100\%$ susceptible population is very expensive and practically a tedious process. On the other hand, everyone cannot take the vaccine as some individuals may have serious allergy and some may have sensitive immune system. In such cases the effect of vaccination may be more adverse than disease or infection. Although, there exists a theoretical threshold proportion of the susceptible individuals by which the epidemic can be prevented by vaccination to this susceptible fraction. This phenomena is known as the herd immunity against a specific infection in a given population. In other words, it refers to the stage in which a given population proportion acquire the immunity against the infection in such a way that infection will not invade the population if it is introduced into it at any random point [13, 25, 125].

Let vaccine be $100\%$ effective and $\rho$ represents the proportion of vaccinated population of susceptible individuals. Then $\rho S(0)$ is the number of susceptible individuals which is removed from the susceptible population and effective susceptible population will be $(1-\rho ) S(0)$. To prevent the disease propagation it is mandatory that effective reproduction number $R_e< 1 \implies \frac{\beta }{\gamma } (1-\rho ) S(0) <1 \implies \rho > \rho _c$ where, $\rho _c = 1- \frac{1}{R_e}$ is known as critical vaccination threshold. A population will acquire the herd immunity if vaccinated population proportion is above this critical vaccination threshold. For example: if values of basic reproduction number $(R_0)$ for a specific infection is 1.5 then approximate value of critical vaccination threshold for this infection will be 33%. This computation is for 100% effective vaccination but if effectiveness of a vaccine is $p \%$ (say) then effective critical vaccination threshold value will be $\frac{\rho _c}{p} \times 100 \% = \frac{33}{60} \times 100 \%= 55 \% .$

### Others mathematical modeling techniques for viral infection

**Fig. 8:**
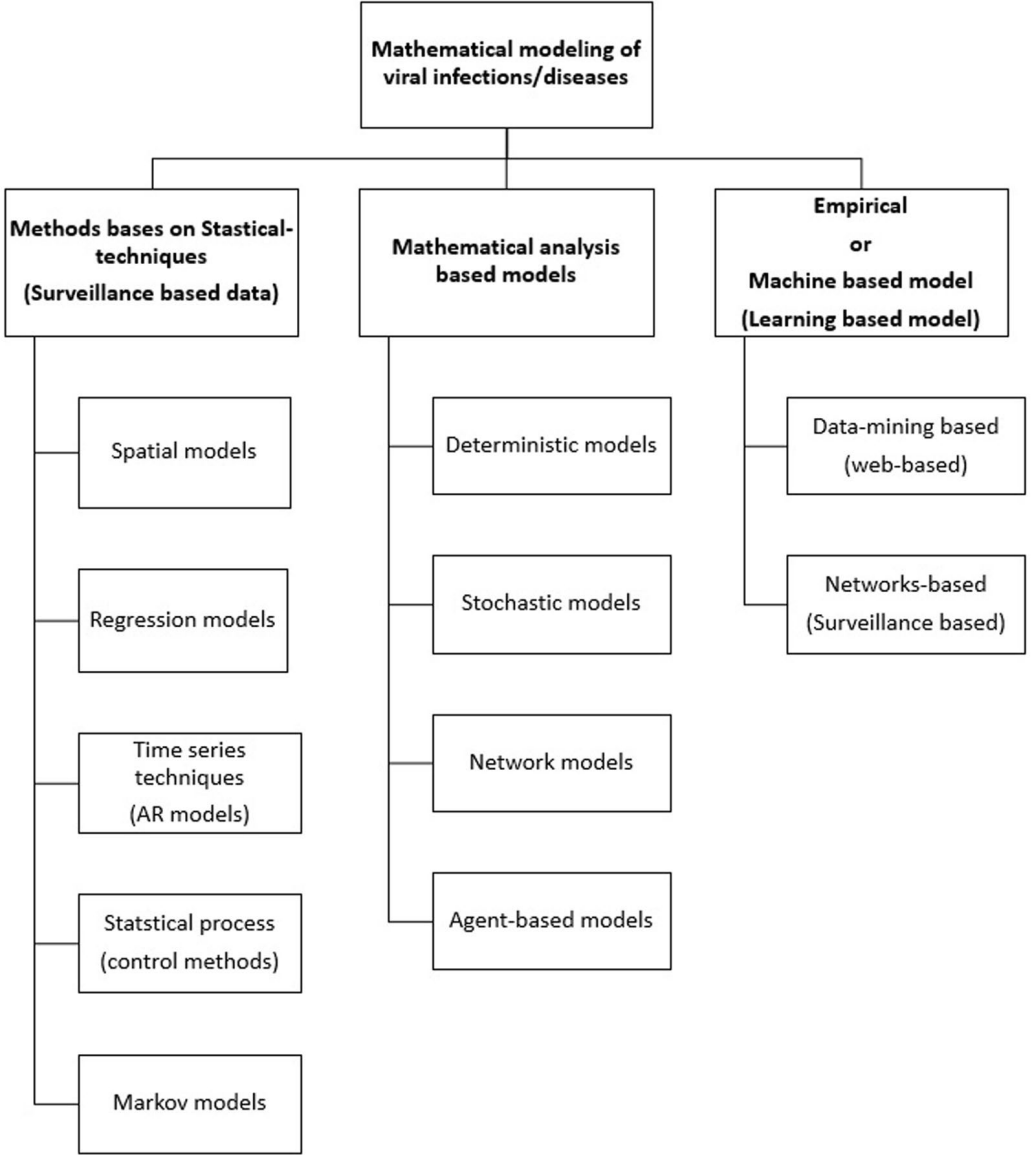
Different techniques used in mathematical modeling of viral infections/diseases

So far, we have discussed the compartmental deterministic mathematical modeling in detail, whereas, there are several different other approaches to investigate the dynamics of viral infections. The types and sub-types of these techniques are shown in Fig. 8. Time-to-time several researchers have worked on the basics of these techniques in the area of mathematical modeling of viral infections, which have been briefly described in the following paragraphs:

#### Statistical-technique based models

Regression techniques [126–131] are very popular among the researchers working in the area of viral infections for predicting and surveillance. These techniques are also used by the Centers for Disease Control in many countries including US, Australia, France and Italy for the investigation of transmission of influenza [132]. Several researchers prefer to use time series analysis based on auto-regressive techniques. Auto-regressive Integrated Moving Average (ARIMA) model, Seasonal Auto-regressive Integrated Moving Average (SARIMA), Neural Network (NN) and Support Vector Machine (SVM) are some examples of auto-regressive techniques [133–137]. Statistical process control methods are also common approach to formulate the mathematical model of viral infections. Two main tools of this techniques are cumulative sum (CUSUM) charts [138–143] and exponentially weighted moving average (EWMA) [144, 145]. CUSUM technique uses a measure of cumulative performance over the time. Whereas, EWMA control chart technique is based on a recursive statistical estimator. Other statistical techniques also lie under this category, such as: temporal scan statistics [146–148]. Hidden Markov models (HMM) [149, 150] are normally used to examine the correlation between time series. These methodologies are useful when we do not know the characteristics of the disease explicitly but able to identify some indicators for the transmission of that particular disease [149, 151]. Spatial models are used to investigate and predict more accurately diseases outbreaks in different locations [152–158]. Spatial models require multivariate methods which are extension of standard uni-variate methods [159]. Abstract techniques like principal component analysis (PCA) is an emerging technique to do this type of analysis. For example Cohen et al. [160] used PCA technique to know the spatial trends of malaria in India. Many other researchers have developed models based on spatial technique to achieve their goals [146, 148, 161–163].

#### Mathematical analysis based models

Mathematical analysis based models can be classified broadly into four categories: deterministic, stochastic, network-based and agent-based. The major difference between deterministic and stochastic modeling is that deterministic models are based on the hypothesis of the mean field approximations whereas in stochastic approach these hypothesis are relaxed. In stochastic modeling approach randomness of individuals has been taken into account. Therefore, in deterministic approach model output is fully determined by the parameter values and initial conditions whereas in stochastic modeling approach same set of parameter values and initial conditions may produce different output for every simulation. Stochastic models may use discrete or continuous-time individual based Markov-chain models [164–166]. More detailed comparison between these two approach is available in Allen et. al. [167]. Time to time different mathematical models for viral infection transmission has been formulated based on stochastic approach. In this series, Lekone et al. [168] formulated the model based SEIR-stochastic modeling for the transmission characteristic of Ebola outbreak in the Democratic Republic of Congo during 1995 . Bishai et al. [169] formulated a SIR stochastic model for transmission dynamics of measles in Uganda. They also estimated the economic burden to eradicate the disease by control policies. Wang et al. [170] used SIR-stochastic model to know the multi-periodic pattern from avian-flu outbreak in North America.

Homogeneous Markov process belonging to the category of Stochastic modeling usually assumed homogeneous and instantaneous interaction between individuals. In complex network models [171–173] these assumptions are relaxed. Dealing with heterogeneity in the contact network is a challenging task in this approach. Heterogeneity in the population exists due to geographical, demo-graphical and economical factors. Therefore topology of the social networks play a significant role in the investigation of the infectious disease transmission in a given human population. In the last two decades, many researchers tried to explain the progression of the diseases on topologically different networks. Kuperman et al. [174] and Reppas et al. [175] used small-world network to explain the dynamics of a simple disease model. Hwang et al. [176] used scale-free network to simulate the disease transmission on it. They tried to find the effect of clustering coefficient and average path length on the disease outbreak. Shirley and Rushton (2005) tried to simulate the disease spreading in different four network structures: Erdős-Rényi, regular lattices, small-world, and scale-free [177]. In this context, many researchers worked on real networks for instance: Read et al. [178] used diary based survey of 3528 individuals for the spread of infectious disease, Christakis and Fowler [179] used 744 students’ contact network at Harvard University to study the influenza outbreak in 2009, Salathé et al. [180] used wireless sensor to construct interaction network among students at an American high school, Kelling et al. [181] used meta-population networks on the basis of 10000 wards in the Great Britan. Rocha et al. [182] used SIR structure on networks to find out the sexual transmitted infection of 50,185 individuals based on data extracted from 12 cities from Brazilian Internet Community.

In agent-based approach, the action and interaction of autonomous individuals or groups (known as agents) is considered. The interaction in complex network may be based on several factors like: transportation facilities in that geographical area, demographic factors, emigration/migration of the individuals and different aspects of transmission between host and pathogens. Many researchers developed their models based on this approach in real-time scenario. In this context, Ferguson et al. (2005) simulated the results for the transmission of H5N1 influenza in southeast Asia [183]. They included the 85 million residing in Thailand and adjoining countries as agents. They also included the demographic parameters and mobility of the individuals in their study. Burke et al. [184] developed an agent-based model for small-pox for a hypothetical township considering 6000–50000 agents. They considered the infrastructure according to US demographic conditions. They also measured the efficiency of various control strategies such as vaccination. Several other researchers developed their simulation tools for these types of modeling such as Episims developed by Eubank et al. [185], GLEam developed by Balcan et al. [186].

#### Machine learning based models

Machine learning based model is a recent technique in which the large data available on Internet or other resources are used to extract the information or trend for prediction perspective. The use of this technique in the analysis of infection dynamics is recent and advanced. In this context, Ginsberg et al. [187] tried for early detection of influenza through Google search queries. They used about 50 million queries for the symptoms of the influenza infection during 2003–2008 for their study. Hulth et al. [188] also worked on influenza (from 2005 to 2007) and data collected via web queries through a specific website. One attempt by Chan et al. [189] has been made to study the transmission dynamics of dengue through the data collected via web queries between 2003 to 2010. The authors collected data from Bolivia, Brazil, India, Indonesia, and Singapore for their study. Policy makers around the world use such mathematical models described here so far to evaluate or develop the intervention policies for existing or emerging infectious disease outbreaks.

## Conclusions

Several studies have shown that mosquitoes transmitted infections have a large percentage in the human population. Therefore it is essential to study the transmission characteristics of mosquitoes transmitted infections. Mathematical modeling is the perfect tool to analyze the situation with minimum resources and time to understand these characteristics. In the article presented above, we reviewed the work done by several researchers/scientists about four viral infections (Dengue, Zika, Chikungunya, and West-Nile). We also discussed the basic concept of mathematical modeling, starting with its origin. The basic $\textit{SIR}$ model has been discussed in detail. More extended models have also been explained and analyzed. Numerical solution techniques to solve these models also have been discussed.

This article provides a piece of sufficient basic knowledge about the mathematical modeling for mosquito-transmitted viral infections (in particular CHIKV, DENV, ZIKV and WNV) for the researchers who wish to start or are working in this field. Moreover, this survey also introduced to modeler for emerging viral infections.

## Abbreviations

SIR: Susceptible-infected-recovered
SEIR: Susceptible-exposed-infected-recovered
